# Looking back and looking ahead: advancing understanding of insomnia economics

**DOI:** 10.1093/sleep/zsae140

**Published:** 2024-06-20

**Authors:** Emerson M Wickwire

**Affiliations:** Division of Pulmonary, Critical Care, and Sleep Medicine, Department of Medicine, University of Maryland School of Medicine, Baltimore, MD, USA; Department of Psychiatry, University of Maryland School of Medicine, Baltimore, MD, USA

In the modern healthcare climate of increasing costs on the one hand and limited resources on the other, understanding the economic aspects of sleep medicine care have never been more important. Key stakeholders including patients, healthcare providers, payers, and employers are keenly attuned to value—including financial costs—and potential benefits of sleep treatments [1]. In turn, these economic considerations directly impact access and reimbursement for sleep medicine care. Indeed, perceived value and potential return on investment (ROI) can be considered second only to clinical effectiveness, in terms of determining how sleep medicine care is delivered in real-world settings.

Consider chronic insomnia, the most common sleep disorder seen in clinical practice. Characterized by persistent difficulty initiating and/or maintaining sleep with the associated daytime consequence, this vexing condition is associated with a broad range of adverse medical (e.g. cardiovascular disease, diabetes, and premature death) and psychiatric outcomes (e.g. depression, anxiety, chronic pain, post-traumatic stress, dementia, and substance abuse), as well as diminished health-related quality of life (HrQOL). Insomnia is also associated with substantial economic costs borne by patients (reduced quality of life), payers (increased direct treatment costs and indirect healthcare resource utilization [HCRU]), employers (diminished workplace productivity and increased risk for costly accidents and errors), and society [2].

Fortunately, effective treatment options exist, including both pharmacotherapies as well as cognitive-behavioral approaches [3, 4]. Cognitive-behavioral therapy for insomnia (CBTI) is widely considered the first-line treatment for insomnia occurring in isolation as well as insomnia comorbid with other sleep, medical, and psychiatric conditions [5, 6]. Relative to insomnia pharmacotherapy, CBTI is equally effective in the short-term, with gains better maintained over time, and is generally associated with a more favorable risk-benefit profile. Even so, CBTI (and insomnia care in general) remains widely underutilized for many reasons, including limited availability and uncertainty regarding potential ROI. Stakeholders including payers and policy-makers want to know, “For every $1 invested in insomnia care for a specific group of patients, what can be expected in return?” [7]

In this issue of *SLEEP*, Fang and colleagues [8] present results of a systematic review and meta-analysis of cost-effectiveness of CBTI. Twelve randomized controlled trials (RCTs) met inclusion criteria and were reviewed in detail. The study was properly registered, guided by a clear protocol, and generally very well-conducted. Overall, this study represents the most significant review contribution to the literature since a comprehensive review of insomnia economics was published in 2015 [2]. Whereas the original review included five studies of CBTI (and five studies of insomnia pharmacotherapy) [2], the study by Fang and colleagues includes a welcome and substantially greater number of studies examining CBTI economics, reflecting increased recognition of the importance of economic considerations among insomnia scientists. As a result of the increased number of studies, Fang and colleagues were able to build upon prior qualitative syntheses [2, 9] and pool data for a quantitative, random-effects meta-analysis, an important contribution to the literature.

Results indicated that relative to various control conditions including waitlist, treatment as usual, sleep hygiene education, and no treatment, CBTI was more cost-effective in terms of reducing insomnia severity and improving HrQOL across time horizons ranging from 8 weeks to 12 months. Notably, relative to control conditions, CBTI was found to increase healthcare costs over 6 months, but differences versus controls were blunted at 12 months; this finding highlights the importance of both treatment costs as well as time horizon when evaluating cost-effectiveness of insomnia care. For example, clinical studies have demonstrated that following CBTI, sleep continues to improve over 24 months [10], with gains maintained at 10 years [11]. Economic analyses that include such extended follow-up durations are lacking.

Notable strengths of the study by Fang and colleagues include adherence to the Consolidated Health Economic Evaluation Reporting Standards 2022 (CHEERS 2022) Statement [12] as well as a number of subgroup analyses, particularly analyses of the cost-effectiveness of digital CBTI (dCBTI) based on five RCTs. Given the potential for dCBTI to increase access to care for the millions of individuals experiencing chronic insomnia, economic evaluation is imperative [13]. In this vein, another important finding of the study by Fang and colleagues is that dCBTI was associated with improved workplace productivity (reduced presenteeism), a key economic outcome from the employer’s perspective. The vast majority of insomnia-related costs are borne by employers, and increasing employee engagement with insomnia care and demonstrating economic value to employers are an essential future direction for the field [14, 15].

At the same time, the current study maintains most central limitations of prior reviews [2, 9] in that included studies are highly heterogeneous in terms of patients studied, comparator groups, economic perspective, economic endpoints (that could lack sensitivity to detect insomnia treatment-related change [7]), and time horizon, to name several factors. Many of these issues are noted by the authors and have previously been considered in detail elsewhere, suggesting important future directions for sleep health economic research. Of particular importance is the need for easily interpretable findings pertaining to ROI for insomnia care in specific populations, which remain elusive. Another gap in knowledge noted by the authors is that insomnia economic data is derived from a small number of countries. Given that a recent literature-based analysis found that approximately 880 million adults (16.6% of adults) worldwide are likely to experiencing insomnia disorder [16], adopting a global impacts of disease perspective can help to engage stakeholders, advance understanding, increase awareness, and guide allocation of scarce health resources worldwide.

The term “value-based sleep” was coined to emphasize the need for sleep scientists and clinicians to define, demonstrate, and maximize the value of sleep medicine care to those whom we serve (e.g. [14, 17],). To this end, Table 1 presents a proposed research agenda and actionable research strategies to advance the science of sleep health economics, updated and adapted to insomnia since prior publication [2, 18]. At a minimum, given the overt and increasing importance of economic issues to all aspects of sleep medicine care delivery, economic outcomes should be incorporated into sleep research studies whenever possible, especially in all RCTs.

**Table 1.** Proposed Research Agenda to Advance Insomnia Economic Science

Sleep health economic domain	Actionable recommendation
Include health economic endpoints	Include measures of direct and indirect costs in insomnia research
Evaluate cost-effectiveness	Include measures of both general and disease-specific measures of HrQOL in future insomnia trials
Study specific populations	Perform health economic analyses among key sociodemographic subgroups including women, older adults, and children, among different ethnic groups, and among patients with varying insomnia severity
Understand comorbid insomnia	Study economic costs of untreated insomnia and insomnia treatments in costly and chronic comorbid disease states
Increase adherence to dCBTI	Study economic cost–benefit of interventions to reduce attrition and increase treatment adherence to dCBTI
Adopt employer perspective	Optimize employee engagement and evaluate cost–benefit of CBTI from employer perspective (i.e. impact on workplace productivity and workplace accident and injury risk)
Evaluate global impact	Evaluate cost-effectiveness of CBTI worldwide in various healthcare delivery systems
Compare economic effectiveness	Comparative economic effectiveness between insomnia treatments to empower stakeholders to make evidence-based decisions regarding allocation of scare healthcare resources

Updated and adapted since prior publications [2, 18]. CBTI, cognitive-behavioral treatment for insomnia; dCBTI, digital cognitive-behavioral treatment for insomnia; HrQOL, health-related quality of life.

In summary, the systematic review and meta-analysis by Fang and colleagues [8] reflects a welcome and important quantitative analysis of the cost-effectiveness of CBTI. The authors are to be commended for this well-conducted study. More important, given the commonality of insomnia and the availability of effective treatments, sleep scientists and clinicians are urged to increase attention to the vital yet understudied scientific domain of sleep health economics.
